# Sustainable Lubrication Methods for the Machining of Titanium Alloys: An Overview

**DOI:** 10.3390/ma12233852

**Published:** 2019-11-22

**Authors:** Enrique García-Martínez, Valentín Miguel, Alberto Martínez-Martínez, María Carmen Manjabacas, Juana Coello

**Affiliations:** 1 High Technical School of Industrial Engineers of Albacete, University of Castilla-La Mancha, 02071 Albacete, Spainmcarmen.manjabacas@uclm.es (M.C.M.); juana.coello@uclm.es (J.C.); 2 Regional Development Institute, Science and Engineering of Materials, University of Castilla La Mancha, 02071 Albacete, Spain; alberto.martinez@uclm.es

**Keywords:** titanium alloys, sustainable lubrication, cryogenic lubrication, MQL

## Abstract

Titanium is one of the most interesting materials in modern manufacturing thanks to its good mechanical properties and light weight. These features make it very attractive for use in the aeronautical and aerospace industries. Important alloys, such as Ti6Al4V, are extensively used. Nevertheless, titanium alloys present several problems in machining processes. Their machinability is poor, affected by low thermal conductivity, which generates very high cutting temperatures and thermal gradients in the cutting tool. Lubricants and cutting fluids have traditionally been used to solve this problem. However, this option is unsustainable as such lubricants represent a risk to the environment and to the health of the operator due to their different chemical components. Therefore, novel, sustainable and green lubrication techniques are necessary. Dry machining is the most sustainable option. Nevertheless, difficult-to-machine materials like titanium alloys cannot be machined under these conditions, leading to very high cutting temperatures and excessive tool wear. This study is intended to describe, analyse and review the non-traditional lubrication techniques developed in turning, drilling and milling processes since 2015, including minimum quantity of lubricant, cryogenic lubrication, minimum quantity of cooling lubrication or high-pressure coolant. The aim is to provide a general overview of the recent advances in each technique for the main machining processes.

## 1. Introduction

Because of their mechanical properties, titanium and titanium alloys are one of the most commonly used materials in manufacturing processes in certain key industries such as the aeronautical, aerospace and medical ones. These alloys exhibit high strength while also being very light [1], and also have very high wear and corrosion resistance. These unique characteristics, along with their possessing the highest strength to weight ratio [2], make these alloys very attractive for such industries. Titanium and titanium alloys offer the best mechanical features possible for applications in which low weight is required.

Nevertheless, pure titanium and titanium alloys present several problems for machining processes, which mean they are classified as difficult-to-machine materials. Their low modulus of elasticity and extreme strength at high temperature [3], and inferior thermal conductivity generate long ductile chips and relatively large contact length between chip and cutting tool in machining processes. Thus, very high temperatures are reached and aggressive thermal gradients appear in the cutting tool. Their low thermal conductivity and high heat capacity play a critical role in the heat dissipation process [4], which finally causes tool wear and a rapid reduction in tool life.

Furthermore, the plastic deformation of the material, friction and high chemical affinity of the titanium and the cutting tool materials produce built up edge (BUE), affecting the geometry of the tool and impairing the surface integrity of the final machined part.

The combination of all these conditions reduces the machinability of titanium alloys, the improvement of which is one of the greatest challenges recently addressed by a large number of researchers. Such researchers are striving to provide new machining strategies for this material, as well as to determine optimal cutting conditions in the machining processes.

Ti6Al4V is the most significant titanium alloy from the point of view of industrial applications. Table 1 shows the distribution of the most frequently used titanium alloys in the literature we have reviewed. Ti6Al4V alloy is estimated to account for 50% of global titanium metal production, and 80% of this corresponds to the aerospace and medical industries [2]. Ti6Al4V is an α−β titanium alloy, in which the amount of beta stabilizer added is about 4–6%. These α−β alloys can obtain different mechanical properties by heat treatment and their characteristics are optimal for application with warm temperatures (400 ${}^{\circ }$C) [5].

Ti553 alloy is a relatively modern alloy, which is gaining prominence in applications in the automotive, chemical and medical industries because of its excellent properties, such as its good strength-to-weight ratio and high hardness at heating state [4]. The machinability of this alloy has not been extensively studied in comparison with Ti6Al4V, and thus the optimal conditions for the machining of this material are still far from determined.

Titanium aluminide (TiAl) is an intermetallic chemical compound of titanium and aluminium as base metals and other elements in small proportions [6]. This material achieves superior properties compared to traditional titanium alloys, such as excellent heat, corrosion and oxidation resistance. Moreover, it is very light, thanks to its high proportion of aluminium. However, it exhibits poor ductility and low fracture toughness [5].

TC17 is an alloy with high toughness with some applications in gas turbine engine components. Althought Ti6Al7Nb alloy has similar properties as Ti6Al4V, no general applications are found with it.

### 1.1. Traditional Lubrication Approach in Titanium Machining Processes

Flood lubrication with abundant quantities of lubricant has traditionally been the focus of attempts to overcome the machinability problems of titanium alloys [7]. The lubricant, coolant and cutting fluids (CFs) industrially used are estimated to account for about 17% of the total manufacturing costs of the final part [8], while the cutting tool costs are only 4% of total machining costs [9]. The cost factor, along with the environmental and health risks associated with the use of these cutting fluids, are the main reasons for attempting to reduce their use. Cutting fluids used as cooling agents contain environmentally hazardous and harmful chemical elements [6] and may cause diseases, such as respiratory problems, asthma and cancer.

The functions of the cutting fluids include lubrication and cooling, which can be carried out in parallel. In short, the objectives pursued by the application of these fluids is to improve the dissipation of the heat generated in the cutting area, which is very high in the machining of titanium alloys (up to 1000 ${}^{\circ }$C), and to reduce the friction between the chip and the cutting tool.

The better or worse performance of the cutting fluid depends on the machining process, as well as the cutting conditions and the fluid characteristics, such as density, viscosity and specific heat. According to Lin et al. [10], the properties of the cutting fluid may affect the cutting conditions when turning Ti6Al4V, depending on the lubrication method. Three main types of cutting fluids exists: mineral, semi-synthetic and synthetic. Synthetic and semi-synthetic fluids are aqueous based fluids in which the good heat conduction of water combined with the oil properties enhances the performance of the cutting fluid.

### 1.2. Need for Sustainability

As previously explained, cutting fluids are damaging for the environment and human health, apart from involving high additional costs in the machining processes. There has been some efforts for using sustainable cutting fluids based on vegetable oils like sesam, coconut, sunflower, palm and others [11]. Some applications of this kind of fluids increases the cutting tool life a 170% in drilling of materials difficult to cut as AISI 316 steel. Coconut oil improves significantly the efficiency of machining processes and with the palm oil better results for Ti6Al4V alloy have been obtained compared to traditional flood lubrication [11,12]. Nevertheless the sustainability of this kind of fluids are controversial nowadays, specially palm oil.

Thus, the next qualitative advance in cutting fluids consists of the use of synthetic fluids made out of a liquid vegetable base and the addition of nanoparticles as Al${}_{2}$O${}_{3}$, MoS${}_{2}$, diamond and graphene [11]. The synthetics fluids reduce the cutting forces and temperature and improve the surface finishing after machining. Moreover, some of these components act in an efficient way in applications in which friction at high pressure appears [13]. Nevertheless the cost of these lubricants is much higher than traditional ones [12].

**Figure 1:**
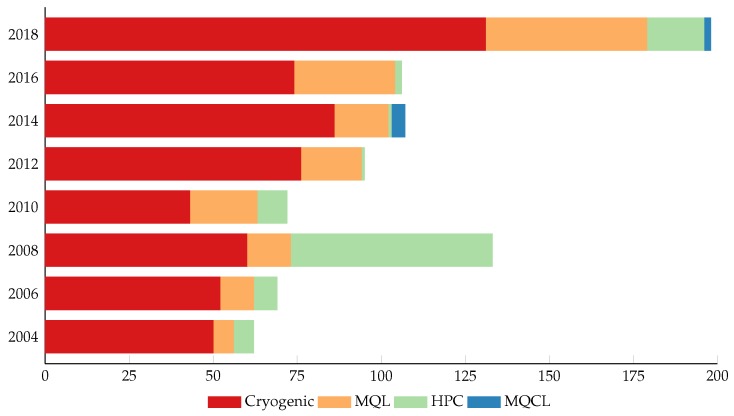
Evolution of principal sustainable machining techniques for titanium alloys.

For these reasons, in recent years, different lubrication and cooling techniques have been developed with the goal of reducing the use of cutting fluids and improving the machinability of titanium alloys under environmentally-friendly conditions [7,12,14,15,16]. Figure 1 shows the evolution of the number of papers related to the principal sustainable lubrication techniques.

These modern techniques provide different solutions for the cooling and lubrication problem, reducing the quantity of lubricant, as in the case of the minimum quantity of lubricant technique (MQL), or substituting the harmful lubricant for another green substance that can provide the cooling action, as in the case of cryogenic lubrication with liquid nitrogen, LN${}_{2}$. This paper reviews the main sustainable techniques, such as minimum quantity of lubricant (MQL), cryogenic lubrication, minimum quantity cooling lubrication (MQCL) and high-pressure cooling (HPC), based on the recent literature since 2015.

Although dry machining can be considered a sustainable technique due to the lack of any lubricant, it cannot be applied in titanium and titanium alloy in most cases because of the high temperatures reached, which cause excessive tool wear and considerably reduce the quality of the machined parts.

The minimum quantity lubrication method (MQL) involves the atomization of cutting fluid droplets mixed in the air and directed at the cutting interface of the tool. The MQL technique is comparatively less hazardous for the environment than flood lubrication because it consumes very little toxic and non-biodegradable cutting fluid and needs less energy to pump the atomized fluid at higher flow rates and pressure [14].

The MQL strategy limits the cutting temperature by reducing the friction force tool-chip during the cutting process, thanks to the effective lubrication directly applied on the rake face of the tool. Compared to traditional flood lubrication, MQL requires much less than 1 L/h of lubricant [17] which is a great decrease compared to over 100 L/h adopted flood lubrication [12], although specific equipment is needed to atomize the lubricant droplets. In addition, vegetable-based oils are extensively used in this lubrication method [8].

Although air contributes to refrigerating the cutting interface and to dissipating heat by using the convective heat transfer mechanism, it has been proven for difficult-to-machine materials, like titanium alloys and nickel-based materials, that the MQL technique is not completely suitable because of its low heat transfer capacity [10] and its role depends on the specific machining process and cutting conditions. Typically, the cooling action of the air is not sufficient to reduce the cutting temperature, being the main drawback of MQL lubrication. Moreover, Mathew et al. [6] demonstrated a low efficiency of the MQL lubrication for deep hole drilling. However, it has been extensively demonstrated that MQL method is able to reduce cutting forces and to improve tool life compared to dry machining, and, depending on cutting conditions, compared to traditional flood lubrication [1]. Mathew et al. [6] reported MQL improvement on Ti aluminide drilling compared to flood lubrication based on cutting forces thanks to better penetration of the lubricant when eliminated chips obstruct its passage. Benjamin et al. [17] explain that the MQL method is able to overcome the formation of a vapor blanket in the cutting zone, which inhibits the effective lubrication effect of flood cooling in operations with low machinability materials.

As a result of the low quantity of lubricant applied in this technique, metallic chips generated during the process are almost dry and can be easily recycled [1].

Definitively, the MQL method solves the friction problem between the chip and the cutting tool by means of effective lubrication with a low quantity of lubricant compared to flood lubrication, which makes it a sustainable, environmentally friendly lubrication technique. Nevertheless, MQL does not enhance the cooling effect efficiently and is unable to rapidly dissipate the heat generated during the machining process.

The MQL approach has been developed to improve the cooling action. Many authors have demonstrated the improvement of mixing oil with water as a mode of transport and cooling source [10]. This technique, called Oil on Water (OoW) MQL, uses water droplets to transport a small quantity of lubricant. Specific equipment is required.

With this improvement, lubricant oil performs the lubrication effect, reducing the friction coefficient between chip and tool, while water, when evaporated, provides a cooling effect, which is more effective than supplied air flow. Briefly, when the water and oil droplet reach the objective surface, water evaporates, decreasing the cutting temperature, and oil remains, forming the lubricant film that reduces the friction force [14].

**Figure 2:**
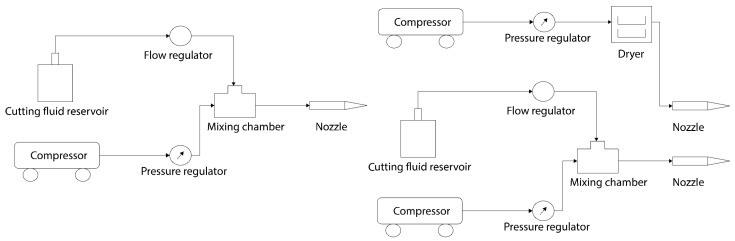
Schematics of MQL and MQCL systems.

Another variant whose purpose is to improve heat dissipation in the MQL method is the minimum quantity of cooling lubrication technique (MQCL) [15]. Figure 2 shows the schematic diagrams for MQL and MQCL set ups. In this method, MQL is combined with sub-zero cooling, provided by low temperature air to enhance heat transfer from the tool-chip interface [17]. This system uses temperatures below 0 ${}^{\circ }$C, but not as low as those achieved in cryogenic lubrication. For both MQL and MQCL procedures, the droplet size of the lubricant must be controlled and always greater than 5–10 μm. If the particles are smaller than that value, they can remain in the air and might cause healthy problems in the machine laborer [15,18]. Pervaiz et al. [8] performed their investigation on turning of Ti6AL5V titanium alloy by using vegetable-oil based lubricant at a flow rate of 60–100 mL/h and supplied air at −4 ${}^{\circ }$C. They found that this MQCL cooling strategy is a successful substitute for conventional flood cooling methods, mostly at high cutting speeds.

Benjamin et al. [17] carried out an investigation on milling of Ti6Al4V alloy under the MQCL approach, using refined palm oil as lubricant at a flow rate of 350 mL/h and a cold temperature of −10 ${}^{\circ }$C. They found surface roughness improvements compared to the MQL technique.

Nevertheless, the optimal conditions of this technique are still under development; Maruda et al. [19] establishes the threshold of useful conditions in terms of droplets diameters and emulsion mass flow and state that the number of droplets that arrives to the part surface are influenced by the volumetric air flow, and the device-part distance. Anyway, some research is being done regarding to improve the performance index of this kind of lubrication, i.e., the use of extreme pressure-antiwear additives [20].

The cryogenic lubrication method has been extensively studied in recent years. In cryogenic lubrication, liquid nitrogen LN${}_{2}$ is usually supplied by means of small-diameter nozzles at a temperature range between −194 ${}^{\circ }$C and −200 ${}^{\circ }$C [16]. The significant cooling action produced is the greatest advantage of this strategy, compared to the MQL method. Cryogenic lubrication is able to effectively control the cutting temperature, reducing the heat generated in the cutting zone, which represents a substantive improvement on the machining of titanium alloys thanks to the high thermal gradients that appear in the machining operations.

Although this technique has been widely studied in the literature on turning, milling and drilling processes, it is not clear under what conditions the improvements are achieved and whether it is economically profitable, due to the expensive cost of liquid nitrogen [15]. The price of liquid nitrogen is a limiting factor for the application of this lubrication technique since large quantities of nitrogen are required, as well as special equipment. Moreover, in many industries, the savings that can be achieved by increasing the life of the cutting insert or reducing the cutting oil is not sufficient compared to the cost of liquid nitrogen. Nonetheless, LN${}_{2}$ is a green lubricant, being harmless for the environment and human health, which makes this technique attractive. A variant of the deployment of liquid nitrogen is the use of dry ice or supercritical CO${}_{2}$ [14]. The differences lie in the temperatures. While liquid nitrogen boils at −178 ${}^{\circ }$C, carbon dioxide boils at −78 ${}^{\circ }$C [21]. In addition, the preservation methods are different. LN${}_{2}$ must be kept at atmospheric pressure and low temperatures in insulated containers, needing specific equipment, while CO${}_{2}$ needs to be preserved at room temperature and high pressure, about 55 bar.

The cooling effect of CO${}_{2}$ is due to the expansion of the gas when it comes out through the nozzle. A pressure drop takes place, resulting in a phase change into liquid and solid due to the Houle-Thompson effect, which promotes the cooling of the cutting area [22].

Shokrani et al. [2] found that cryogenic lubrication with LN${}_{2}$ on the milling of the Ti6Al4V alloy reduced surface roughness by 40% in comparison to flood cooling, while tool life incremented almost three times. Nevertheless, Dix et al. [23] found, using the Finite Element Method (FEM), that cryogenic cooling produced high torque and force in the axial direction on drilling in comparison to dry machining.

Bordin et al. [24], reported that cryogenic cooling is the optimal solution and most suitable method for machining titanium parts in case of medical applications, because it minimizes the further severe cleaning requirements thanks to the lack of oil lubricant.

High-pressure cooling (HPC) is a technique whose principle is not based on the reduction of the quantity of lubricant, but on its efficient use, through effective penetration in the contact zone between the tool and the chip and in the flank face. In comparison with flood cooling, in which the average flow rate of lubricant is about 1.7 L/min [12], HPC utilizes a greater quantity of coolant. Ezugwu et al. [25] performed their study by using flow rates between 18.5 L/min and 24 L/min, while Mia et al. [26] utilized a flow rate of 6 L/min at a pressure of 8 MPa. For these reasons, HPC should not be classified as a green, environmentally friendly lubrication technique.

Laser-assisted machining is a thermally assisted machining process in which the method of operation is notably different from cryogenic lubrication and which has been gaining popularity in recent years [27]. In this modern technique, the specific area of the workpiece is preheated before the cutting process to reduce the flow stress and enable chip formation. This method, effective for machining processes of difficult-to-cut materials, such as titanium alloys, requires specific equipment and high precision in the control of laser variables [28]. The power of the laser and its movement velocity are critical parameters to promote improvements in the reduction of the cutting force. Nevertheless, diffusion can be increased thanks to the temperature reached by the material (about 500 ${}^{\circ }$C). Therefore, the wear processes involved can be accelerated. In addition, thermal shocks can be produced by the temporal delay existing between the material overheated and the arrival of the cutting tool. Tool damage would appear if the laser variables are not properlly controlled [29].

## 2. Literature Review Methodology

This study is strictly focused on the machining processes of titanium and titanium alloys, considering the importance of these materials in current industrial manufacturing processes. The main objective of this review is to analyse the state of the art of the principal modern lubrication techniques that have been widely developed over approximately the last 20 years.

The literature includes many reviews on these lubrication techniques. However, they either give a limited explanation of each method or focus on only one of them, analysing a number of studies on one machining process.

The purpose of this paper is to provide firstly a general overview of each technique, and then a detailed analysis of each one, addressing the principal machining processes (turning, milling and drilling), based on the most novel works found in the published literature, and always with regard to titanium and titanium alloys. For this reason, this work review s from 2015 to 2019. It has been conducted using the Web of Science search engine.

**Figure 3:**
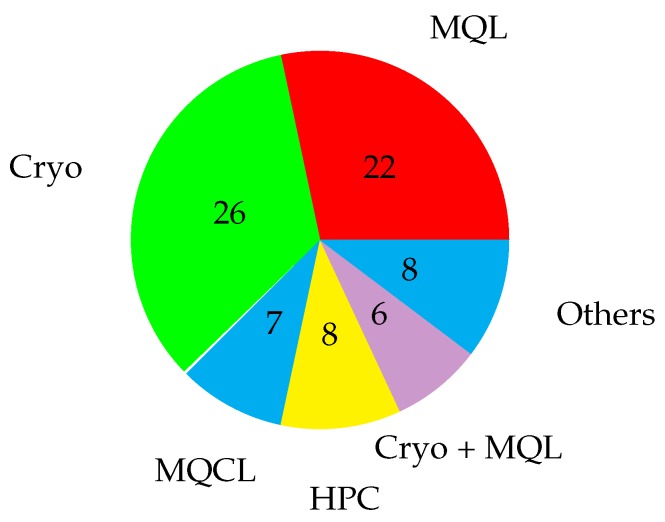
Number of papers on different techniques. Minimum quantity of lubricant (MQL), cryogenics (Cryo), high pressure coolant (HPC), minimum quantity of cooling lubrication (MQCL).

Machining has been used as the general topic for the research. Terms such as MQL, HPC, cryogenic, MQCL, or the same terms written in full have been included in the search for papers related to each technique and filtering from the year 2015. All the papers reviewed are classified in Table 2, according to the type of process, material used, technique evaluated, number of citations and impact of the journal. The number of papers for each technique is shown in Figure 3.

Cryogenic and minimum quantity of lubricant are the two most extensively studied techniques, while high-pressure cooling is only used in turning processes. Other techniques, such as dry machining, ultrasonic vibration assisted machining, machining with specific inserts or electrostatic high-velocity solid lubricant machining have been classified together.

A statistical analysis of these papers has been developed with the aim of obtaining the main parameters studied for each of the principal machining processes, such as cutting forces, surface integrity, cutting temperature, tool wear, chip morphology, friction, specific energy or power consumption. The principal variables studied in each paper on drilling, milling and turning are shown in Table 3, Table 4 and Table 5.

**Figure 4:**
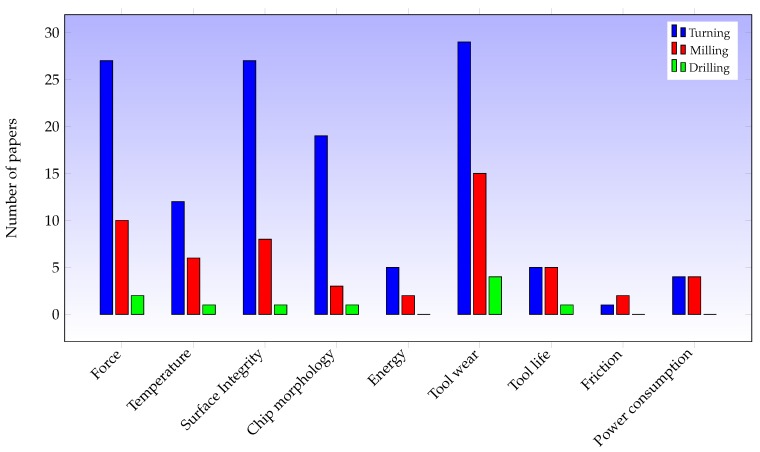
Main criteria mentioned in papers on turning, milling and drilling.

Table 3, Table 4 and Table 5 reveal that the most commonly researched aspects are cutting forces, tool wear and surface integrity of the machined parts, while parameters such as friction coefficient or power consumption are rarely analysed, Figure 4. Cutting temperature is an important variable that is the subject of less research than cutting forces or tool wear due to the difficulty of its being accurately measured in the cutting area.

With the aim of providing a more detailed overview of the state of the art, the highest-ranked papers on drilling, milling and turning have been filtered, firstly taken into account the number of citations and the impact index of the journal. In this way, a number around 15 papers have been managed for turning and milling processes; for drilling, all papers have been carefully analysed as the few number of total papers published. After that, a second filtering step has been applied based on a detailed reading, selecting a balanced quantity of papers about each main lubrication technique. This led to take into account eight manuscripts for turning and milling processes.

For the selection of these papers, a content analysis has been made that considers the influence of the lubrication process on the technological capacity of the processes in terms of surface roughness, cutting forces, temperature and tool life. In addition, it has been evaluated qualitatively the discussion and conclusions carried out by the own authors in comparison with traditional lubrication.

The main strenghts of the review methodology applied herein are the relevant and complete classification of involved papers based on the machining process, the lubrication technique used and the different variables analysed and researched by the authors. This permits to locate in an easy way the points of interest of each paper to be considered as background for specific researchings. Likewise, based on some representative papers, the different lubrication techniques and their effect have been carefully analysed providing the grounded fundamentals of them.

Nevertheless, the methodology is conditioned to the use of a single search engine that, althought is the most important one, might set aside some significant papers. Besides, sometimes, the keywords in the papers could not be well identified according to our searching criteria.

### 2.1. Sustainability in Milling Processes

A detailed review of the most significant sustainable techniques in milling processes was conducted. The papers selected are shown in Table 6.

With this selection of milling research papers, a general overview can be obtained about the state of art of each of the main sustainable techniques.

Shokrani et al. [2] investigated the application of cryogenic lubrication with liquid nitrogen LN${}_{2}$ in the milling process of Ti6Al4V alloy, compared to dry and flood lubrication. They performed the experimental investigation by supplying cryogenic nitrogen around the cutting tool at −197 ${}^{\circ }$C, 1.5 bar of pressure and with a flow rate of 0.4 L/min, but without submerging the workpiece in LN${}_{2}$. For the experiments, they used a TiN-TiAlN coated solid carbide end mill and tested three cutting velocities (30 m/min, 115 m/min and 200 m/min), three feed rates (0.03 mm/tooth, 0.065 mm/tooth, and 0.1 mm/tooth) and three depths of cut (1 mm, 3 mm and 5 mm).

This study examined the potential of the cryogenic technique to improve surface roughness and tool life, thanks to decelerating thermally induced wear, while reducing power and energy consumption. The authors found that, on average, surface roughness was 30.42% lower in comparison with dry and flood machining. They also found that the environment (dry, flood or cryogenic) has a 21.5% contribution on final surface roughness and a 73% contribution in power consumption.

Park et al. [1] obtained poor results for the application of only cryogenic LN${}_{2}$, both by the internal and external method. The cutting force at the beginning of the process was lower than for flood lubrication, but a significant increase was obtained at the final pass. In addition, the criterion of tool rupture was achieved in both cases. The reduction of tool life is explained by the lack of effective lubrication due to the deep axial depth of cut with a large contact area between the tool and chip, causing excessive adhesion on the tool.

These authors also investigated the end milling process of Ti6Al4V alloy, comparing different techniques, such as flood coolant lubrication, MQL mixture with Hexagonal Boron Nitride nano-particles and the combination of internal cryogenic and Nano-MQL. They conducted the experiments at cutting speeds of 72 m/min for flood lubrication and 86 m/min for the other techniques, feed rate of 0.1 mm/tooth and depth of cut of 24.5 mm. They used a tool coated with Aluminium Chromium Nitride (AlCrN).

They found that the application of Nano-MQL always reduces the cutting forces in comparison with flood cooling, obtaining the best results for the combination of Nano-MQL and internal cryogenic. In addition, tool life was enhanced by up to 32%, the reasons being the effective lubrication supplied by Nano-MQL and the reduction of cutting temperature thanks to cryogenic lubrication.

In another study, Park et al. [9] analysed face milling and end milling of Ti6Al4V alloy under dry, flood and MQL. They used an uncoated insert and the conditions for the face milling experiments were cutting speed of 47.7 m/min, 76.4 m/min, 100 m/min and 120 m/min, feed rate of 0.15 mm/rev and depth of cut of 2 mm. For the end milling experiments, the cutting speeds were 72 m/min and 90 m/min, the feed rate was 0.1 mm/rev and the depth of cut was 1.5 mm. They tested two MQL methods: the application of a vegetable oil at the tool-chip interface at 5 bars and 3 mL/min and MQL mixture with exfoliated graphite nano-platelets (MQLN). They showed that MQLN is most effective at a high cutting speed because nano-particles play the role of a lubricant reducing the friction between the tool and work material.

They found MQL reduced cutting force compared to dry and flood machining in all cases, obtaining the best performance in combination with cryogenic conditions.

Similar results were obtained by Tapoglou et al. [22]. These authors reported that the best performing cryogenic method was CO${}_{2}$ in combination with the MQL technique, being better than MQL and the combination of CO${}_{2}$ and air when they used a single insert. When they used five inserts, they found that at 70 m/min and 80 m/min, the combination of MQL and cryogenic CO${}_{2}$ improved tool life by up to 29% and 32%, respectively, compared to MQL. Nevertheless, the application of cryogenic CO${}_{2}$ at maximum velocity reduced tool life in comparison with the MQL technique, thus not being an efficient lubrication method.

Similarly, Benjamin et al. [17] focused their study on comparing MQL and MQL with sub-zero cooling on end milling of Ti6Al4V alloy. They used refined palm oil as lubricant with a flow rate of 350 mL/h and a pressure of 2 bar. The cold stream was supplied by a Vortex tube at −10 ${}^{\circ }$C and 6 bar. The cutting conditions were cutting speed between 90 m/min and 150 m/min and feed rate from 0.025 mm/rev to 0.075 mm/rev.

They found that lower temperatures were achieved under the MQCL technique in comparison with MQL with cold air, which increased the viscosity of the palm oil, improving its lubricating properties.

In addition, with MQCL, the tool life was increased from 11.06 min to 15.9 min and flank wear decreased by 19%.

Park et al. [9] examined what increase in flow rate is needed in the MQL technique as cutting velocity increases. They showed that an optimal flow rate exists for each cutting condition that improves tool life.

Celik et al. [34] studied the influence of cryogenic treatment of WC-Co end mills for machining Ti6Al4V alloy. They analysed the behaviour of coated and uncoated cryogenically treated end mills over 12 h, 24 h and 36 h, developing the experimental tests under dry condition. They found that 36 h cryogenically treatment reduced the friction coefficient and friction forces. In the milling process, an increment of cutting forces was demonstrated in each pass due to toll wear. Increasing the cryogenic treatment times from 12 to 36 h positively affected the coated and uncoated tools, except for the AlTiN sample, in which the coating substrate interfacial adhesion bond was weakened.

AlCrN coated tools with 36 h of cryogenic treatment showed the best performance for both tool wear and cutting forces, while AlTiN coated tools exhibited lower performance with the treatment. In conclusion, it can be said that cryogenic treatment is not an effective method to improve milling process performance when working with an AlTiN coated tool.

Bermhingham et al. [27] studied tool life and wear in laser assisted milling of Ti6Al4V. They performed their face milling experiments under dry conditions, MQL, flood coolant, LAM and a combination of LAM with MQL with the aim of analysing the influence of the environment in tool life. Using a PVD coated (TiN) mill, they tested three laser power levels (50 W, 100 W and 150 W) to analyse the influence of overheating the workpiece material.

They found that laser-assisted milling increased tool life at some cutting speeds and decreased it at others. LAM at high power (150 W) was found to produce the lowest tool life of any test, which means that overheating reduces tool performance due to thermal shock. Compared to the rest of lubrication techniques, laser-assisted milling generated an improvement in tool life at low velocity. For the combination of LAM with MQL, no failure appeared at 69 m/min after 28 min of testing. It was verified that for high cutting velocities, laser-assisted milling has a detrimental effect on tool life due to thermal shock, while the combination of LAM with MQL is capable of improving tool life as the cooling action of MQL slows the rate of thermal wear processes.

Sim et al. [29] analysed the optimal laser power and inclination angle for laser-assisted milling of Ti6Al4V alloy. Using FEM, they studied the influence of the milling rotation angle in relation to the inclination angle of the workpiece surface with respect to the horizontal surface. They found that for inclination angles greater than 30${}^{\circ }$, the preheated temperature decreased in line with the increase of the inclination angle in the tool path until 75${}^{\circ }$, from which a new increment took place, meaning that the minimum value occurs for 75${}^{\circ }$.

In the experimental tests, they found that the cutting force decreased depending on the increase in the path inclination angle. They showed that the preheating temperature decreased as the tool path inclination angle increased, but the preheating temperature increased when the tool path inclination angle was 75${}^{\circ }$ or more, which corresponds to the simulated results.

### 2.2. Sustainability in Turning Processes

There now follows a detailed review of the most important sustainable techniques in milling processes. The papers selected are shown in Table 7.

This selection of papers on turning provides a general overview on the state of the art of each principal sustainable technique.

Mia et al. [3] performed their experimental study on turning of Ti6Al4V alloy by applying high-pressure coolant technique on the flank and rake surfaces of the tool. They compared the difference in surface roughness and tool wear between machining at dry and HPC conditions, using a coated carbide tool (TiN, WC and Co).

They found that double jet action significantly improved the heat transfer because the coolant is able to reach the point of highest temperature by overcoming the chip obstruction and removing it from the cutting zone. Using ANOVA analysis, they found that the environment lubrication has an 18% influence on the final surface roughness. HPC reduced the contact length between chip and tool and inhibited the probability of BUE formation, improving the tribological interaction. Similar results were obtained by Busch et al. [21].

Nevertheless, although HPC decreases the surface roughness at high cutting speed, at low cutting speed the effect was contrary, due to the sliding of chips over the tool surfaces. If coolant output pressure is not sufficient to break the chip and cutting velocity is low, the adhesiveness of the chip is enhanced.

Pervaiz et al. [8] studied the effect of minimum quantity cooling lubrication (MQCL) on Ti6Al4V turning. They used a vegetable oil-based MQL system at flow rates between 60 mL/h and 100 mL/h. The cooling action was added by pressurized air at 0.5 MPa and −4 ${}^{\circ }$C and an uncoated cutting insert was used.

It was found that the application of different oil flow rates under MQCL had no impact on surface roughness. At 90 m/min and lower feed levels, the cutting force showed a decreasing trend with the increment of lubricant flow rate due to effective lubrication. Nevertheless, at the highest feed level, a higher flow rate increased the cutting force. Pervaiz et al. suggested that increased cooling of the workpiece helps maintain material hardness without thermal softening. Similar conclusions were reached by Lin et al. [10] when they analysed the turning process of Ti6Al4V under MQCL conditions. They found that although the lowest air temperature of −26 ${}^{\circ }$C provided a better cooling effect by reducing the cutting temperature, the lowest cutting forces and surface roughness were obtained for −16 ${}^{\circ }$C of temperature. This is due to the reduction of Ti6Al4V temperature that enharden the material. However, both temperatures provided better results than for dry and wet machining conditions.

Sartori et al. [48] conducted their research on semi-finishing turning of Ti6Al4V ELI using cooled gaseous nitrogen, comparing its effects to wet condition and cryogenic liquid nitrogen lubrication. The cutting parameters were cutting speed of 80 m/min, feed rate of 0.2 mm/rev and depth of cut of 0.25 mm. A TiAlN coated tungsten carbide tool was used. Gaseous nitrogen was applied at 2.5 bar in a range of temperatures between 0 ${}^{\circ }$C and −150 ${}^{\circ }$C. For the wet condition, a water emulsion with 5% of semi-synthetic cutting fluid was utilized. For cryogenic tests, liquid nitrogen was supplied at 15 bar and −196 ${}^{\circ }$C.

They found that LN${}_{2}$ lubrication increased flank wear by 30% with respect to the wet condition, while cooled N2 always improved tool life. The best results took place at −100 ${}^{\circ }$C, with a reduction of flank wear of 26% and 43% with respect to wet and LN${}_{2}$ cooling. Less difference was found with an N2 temperature between 0 ${}^{\circ }$C and −100 ${}^{\circ }$C, which does not justify the cost involved in reducing the temperature. Crater wear was considerably reduced by N2 at −75 ${}^{\circ }$C, while the greatest reduction took place at −150 ${}^{\circ }$C. The lowest tool wear was found at −100 ${}^{\circ }$C, with a reduction of 43% compared to LN${}_{2}$, for which the thermal power required to achieve a low temperature is almost 10 times the thermal power required by gaseous nitrogen. However, the improvements were already evident from −50 ${}^{\circ }$C.

Liquid nitrogen was found to ensure the best surface roughness of the machined part. However, N2 conditions at −100 ${}^{\circ }$C and −150 ${}^{\circ }$C were also close to this result.

Nevertheless, good results were obtained by Krishnamurthy et al. [49] in their study. They found that cryogenic treatment allowed a reduction of 300 N in cutting forces in comparison with dry machining, which corresponds to a 25% reduction. During the cryogenic treatment, chip segments became susceptible to fracture, which was detected by the segmentation of the chip. Using a Charpy V-notch impact test, they found that less energy is needed to break Ti6Al4V alloy at cryogenic conditions. Nevertheless, in these conditions, the shear strain of the machined piece was lower, which is an indicator of the high flow stress of Ti6Al4V at this temperature.

Krishnamurthy et al. [49] performed turning of Ti6Al4V alloy by cryogenic cooling and using ethanol in metal removal fluid (MRF). They performed turning tests under different conditions, such as dry machining, flooded machining using water-based an MRF mixture with vegetable oil and flooded machining using the same MRF with an addition of 10% of volume of ethanol, both with a flow rate of 8.3 mL/s. Finally, they performed dry machining after having immersed Ti6Al4V workpieces in a bath of LN${}_{2}$ for 20 min.

Applying ethanol, a further reduction of 65% in cutting forces was generated and the best roughness surface was obtained, thanks to a reduction in the coefficient of friction at the tool-workpiece interface.

Mia et al. [45], in other different study, evaluated surface roughness and cutting forces in the turning of Ti6Al4V under cryogenic lubrication, applied at flank and rake faces and using two cutting inserts (SNMM 120408 and SNMG 1240408).

Using an ANOVA, they found that, under cryogenic conditions, the greatest variation in cutting force came from the cutting velocity, followed by the feed rate, while the tool showed an insignificant impact. For the surface roughness, the variations came from the cutting tool, SNMM insert, associated with lower cutting forces, producing the lowest surface roughness with a reduction of 50% in comparison with the other insert.

Gupta et al. [47] studied the turning process of Ti grade 2 under MQL conditions in comparison with dry machining. By using Box-Behnkens response surface methodology and ANOVA, they obtained the most influent parameters in the machining process. The turning tests were conducted under a cutting velocity from 200 m/min to 300 m/min, feed rate from 0.1 mm/rev to 0.2 mm/rev, depth of cut of 1 mm and approach angle from 60${}^{\circ }$ to 90${}^{\circ }$, by using a CBN insert, coated with TiN. For MQL conditions, the flow rate was fixed at 300 mL/h, with an output pressure of 5 bar.

They verified that with the MQL lubrication condition, lower cutting temperatures were achieved due to effective lubrication, producing lower forces than for dry cutting, for which the no lubrication condition resulted in greater chip-tool contact length. Gupta et al. found that an increase of the cutting speed from 200 m/min to 250 m/min was beneficial and cutting forces, surface roughness and tool wear were reduced thanks to an initial increase in cutting temperature, which is able to soften the workpiece. However, the increment of cutting velocity from 250 m/min to 300 m/min, generated a negative effect on cutting forces, tool wear and surface roughness because MQL lubrication does not sufficient cooling capacity to evacuate heat.

Lin et al. [10] analysed the turning process of Ti6Al4V under the oils on water MQL and the MQCL approach. Oils on water (OoW) is a lubrication method in which a thin layer of oil is transported by droplets of water, providing effective lubrication and cooling effect. They tested two forms of spraying the droplets, from the interior of the cutting tool with a specific insert (IOoW) and from the outside of the cutting tool (EOoW). Based on chip morphology, cutting temperatures, forces, surface roughness and tool wear, they analysed the differences between OoW techniques and MQCL, for which MQL was mixed with cryogenic air between −16 ${}^{\circ }$C and −26 ${}^{\circ }$C.

Under EOoW conditions, the effects of three different spraying locations (rake face, flank face, and rake and flank faces) were studied. Under IOoW experiments, the effects of small (1.2 L/h) and large (2.4 L/h) amounts of water were studied. Two different lubricants were used, fatty alcohol and synthetic ester, with different lubricity and cooling abilities. The turning tests were conducted under a cutting speed of 70 m/min, 90 m/min and 110 m/min, feed rate of 0.25 mm/rev and depth of cut of 1 mm, using coated carbide inserts.

From these EOoW turning tests, they found the lowest forces for flank face spraying location because the chips were much shorter due to the air direction of the shingle. Nevertheless, the highest temperature was achieved for this configuration due to the lack of lubricant on the rake face. The lowest temperature was obtained from the double air direction. EOoW on the flank face provided the best surface roughness thanks to better chip breaking.

In the case of internal oil on water, it was found that the larger amount of lubrication reduced cutting forces and cutting temperatures thanks to effective cooling capacity. However, poorer surface roughness and greater tool wear was obtained in comparison with the lower quantity of lubricant. Lin et al. suggested the explanation that when a small amount of water is used, the mixture of oil and water takes the desirable configuration, oil forms a thin layer around the water droplet and provides an effective lubrication. In the case of using a large amount of oil, the configuration is inverse. Water forms a thin layer around the oil droplets, reducing the lubrication properties.

In addition, Lin et al. reported that lubricant properties had an influence on the IOoW method but not on the EOoW method.

### 2.3. Sustainability in Drilling Processes

A detailed review of the main sustainable techniques in milling processes was conducted. The papers selected are shown in Table 8.

This selection of turning papers provides a general overview on the state of art of each principal sustainable technique.

Perçin et al. [30] studied the effect of MQL and cryogenic lubrication on micro-drilling processes of Ti6Al4V. They compared cutting parameters, such as cutting forces, torque or tool wear, by developing micro-drilling tests under dry, wet, MQL and cryogenic conditions. The experiments were conducted under five different spindle speeds and five feed rates. The depth of the holes was established as a fixed 3 mm. They used a 700 μm diameter uncoated tungsten carbide micro-drill. For the MQL test, lubricant was supplied under low pressure, lower than 3 bar, because higher pressures broke the tool.

They found that, under cryogenic conditions, the cutting force was always greater than for dry and wet conditions, while under MQL, for the greatest feed rates, the force was lower than for the dry condition. The improvement was also seen for the lowest cutting speed in comparison with dry cutting.

The use of lubrication reduced cutting force and torque. At the beginning of the hole, torque was higher for wet and MQL lubrication because the lubricant made it more difficult for the chip to be released. However, with the cutting time, wet and MQL conditions provided better performance in the drilling process due to effective lubrication reducing the torque.

Nevertheless, the best quality for surface roughness was obtained for both wet and MQL lubrication. The results for the cryogenic conditions were worse than those for the dry condition. In addition, they found that the increment of cutting speed reduced surface micro-hardness due to thermal softening. Cryogenic lubrication conditions reduced softening, obtaining greater hardness. For this reason, cryogenic lubrication provided the greatest surface micro-hardness. It also ensured the minimum tool wear, while MQL generated less wear compared to dry cutting due to less abrasion.

Mathew et al. [32] investigated the drilling process of titanium aluminide under MQL lubrication. The experiments were conducted under a cutting speed of 72 m/min and feed rate of 0.01 mm/rev with a depth of cut of 10 mm (low aspect ratio) and 37.5 mm (high aspect ratio). A 4 mm diameter solid carbide drill (TiAlN coated) was used.

They found that in the high aspect ratio (HAR) method, in the absence of coolant for dry cutting, the temperature generated on the workpiece and the cutting tool was significantly higher than for MQL lubrication. This means that MQL provided effective lubrication to evacuate the heat.

Built-up edge was formed in both cases, but, for the MQL condition, there was found to be less BUE formation due to the reduction of chip adhesion on the tool. During LAR tests, the probability of BUE forming is reduced. Mathew et al. analysed tool wear based on tool roughness after 4 holes, comparing it to the initial roughness, reporting that MQL lubrication reduces tool wear significantly, providing as good results as wet machining. Similar results were presented by Nandgaonkar et al. [31]. They developed their experimental investigation on drilling Ti6Al4V alloy under oil on water MQL approach by using a 8 mm diameter solid carbide twist drill coated with TiAlN, finding that 43 holes of 20 mm depth could be made under dry conditions, while, under oil on water MQL conditions, 55 holes could be completed. This means that tool life was improved by 27% thanks to effective lubrication which reduces chip-tool contact length and cutting temperature. The authors reported that tool wear was reduced by 66% under oil on water MQL conditions.

In another study in the same experimental conditions [6], Mathew et al. analysed the evolution of cutting force and torque on drilling titanium aluminide at different aspect ratios. They found that for low aspect ratio (LAR), MQL lubrication provided a reduction in cutting force compared to wet lubrication and dry machining. Torque under MQL and wet conditions was always lower and more stable than under dry drilling.

However, different results were obtained for HAR drilling. In this case, the thrust force achieved under wet conditions was lower than for MQL lubrication, mainly at the end of the drilling process. The authors explained that for HAR drilling the air pressure is not sufficient to evacuate the chip, in addition to the amount of lubricant being insufficient to ensure the evacuation of heat, which causes higher tool wear.

In conclusion, these papers propose MQL as an efficient solution for drilling titanium aluminide at low aspect ratio, while it must be optimized for high aspect ratio drilling, for which the performance index is lower than for wet lubrication.

## 3. Conclusions

Titanium and titanium alloys are widely used in industry for aeronautical and biomedical applications, thanks to their good mechanical properties and low density. Ti6Al4V is the most important alloy, representing 50% of titanium production, although in recent years interest has been growing in titanium aluminides. Based on the literature review, the following conclusions can be drawn:

- Due to the low modulus of elasticity and thermal conductivity, as well as the high chemical affinity with the cutting inserts, the machinability of titanium alloys is extremely poor, exhibiting several difficulties for the machining processes.
- Sustainable lubrication methods are necessary to replace flood lubrication in machining processes, so that machinability is increased and is more environmentally friendly. Techniques such as cryogenic lubrication, minimum quantity of lubricant (MQL), minimum quantity cooling lubrication (MQCL) or laser-beam assisted machining (LBM) have been analysed, focusing on turning, milling and drilling processes.
- The cryogenic lubrication technique has been found to be a potential substitute for traditional flood lubrication. Many authors have concluded that it is an effective method of reducing surface roughness on machined parts, obtaining significant improvements compared to flood lubrication, up to 65% on turning processes [49]. Nevertheless, no clear conclusions have been reached on the performance of LN${}_{2}$ cryogenic lubrication when tool wear and tool life is analysed. A number of authors have obtained tool life improvements under certain cutting conditions on milling and turning processes, but many others have found that tool wear is greatly increased under cryogenic conditions due to the increment of titanium hardness at low temperatures, which also increases the cutting force compared to those obtained under traditional lubrication conditions, although they are smaller than for dry machining. A solution to the hardness increment proposed by various authors is cryogenic treatment of the tool before machining, without affecting the part to be machined, obtaining good results.
- The minimum quantity of lubrication (MQL) method has been proposed by many authors as a good solution to reduce oil quantity in machining processes, achieving the same or better results as for flood lubrication. Cutting forces in milling and turning processes can be reduced by using the MQL technique, although for drilling processes, it has been found to have a great dependence of the cutting conditions and hole aspect ratios, because the difficulty of eliminating the chips in deep hole drilling increases the forces. For low aspect ratios, increments up to 27% on tool life have been obtained on drilling processes [32].
- The minimum quantity cooling lubrication (MQCL) technique has been found to be a good combination of MQL and low temperature supplement. It can efficiently combine the friction reduction effect of MQL and temperature reduction effect of the cooling agent. The application of cooling air or gaseous N2 at temperatures from −4 ${}^{\circ }$C to −26 ${}^{\circ }$C has been found to be a good solution to reduce cutting forces and surface integrity on turning processes. Nevertheless, the combination of MQL and cryogenic LN${}_{2}$ has been found to be a less efficient method than MQL, due to increased cutting forces.
- Laser-beam assisted machining (LAM) has been suggested as a potential method for reducing cutting forces in milling operations, thanks to decreased titanium flow stress. Nevertheless, the process parameters are difficult to control and the use of a high power laser has been shown to cause excessive wear on the tool. Some authors have combined LBM with MQL with good results.

Finally, it has been found that the most widely studied criteria for the analysis of machining processes are cutting forces, surface roughness and tool wear, but important phenomena, such as friction, have not been sufficiently studied. For this reason, future research should focus its efforts on the characterisation of the friction phenomenon between the tool and the chip, which is closely related to cutting process performance.

In addition, the analysis should be extended to other materials that are gaining prominence in applications within the industrial sector, such as titanium aluminides, for which the machining processes have been the subject of limited study, especially under environmentally sustainable working conditions.

## Figures and Tables

**Table 1:** Proportion and composition of titanium and titanium alloys in the reviewed literature.

Material	Number of Studies	Proportion (%)	Composition (%)							
			Al	V	Mo	Cr	Fe	Sn	Zr	Nb
Titanium	3	5.08	-	-	-	-	-	-	-	-
Ti6Al4V	49	83.05	6	4			0.25			
Ti5553	3	5.08	5	5	5	3	0.5			
TC17	1	1.69	5		4	4		2	2	
Ti6Al7Nb	1	1.69	6							7
Ti aluminide	2	3.39								

**Table 2:** Classification of reviewed literature (June 2019).

Reference	Year of Publication	Studied Material	Machining Process	Analysed Lubrication Technique	Number of Citations	Impact
[6]	2017	Ti aluminide	Drilling	MQL	9	1
[30]	2016	Ti6Al4V	Drilling	Cryogenic/MQL	9	1
[31]	2016	Ti6Al4V	Drilling	Oil on water MQL	0	
[32]	2018	Ti aluminide	Drilling	MQL	0	2
[1]	2015	Ti6Al4V	Milling	Cryogenic/MQL/Cryogenic + MQL	15	3
[2]	2016	Ti6Al4V	Milling	Cryogenic LN${}_{2}$	7	3
[8]	2017	Ti6Al4V	Milling	Cryogenic/MQL/Cryogenic + MQL	12	3
[17]	2017	Ti6Al4V	Milling	MQL and MQCL	5	1
[22]	2017	Ti6Al4V	Milling	Cryogenic CO${}_{2}$/MQL/Cryogenic + MQL	4	
[27]	2015	Ti6Al4V	Milling	Laser-assisted	27	1
[29]	2016	Ti6Al4V	Milling	Laser-assisted	10	2
[33]	2015	Ti6Al4V	Milling	Laser-assisted	5	2
[34]	2017	Ti6Al4V	Milling	Cryogenic	3	2
[35]	2017	Ti6Al4V	Milling	Cryogenic LN${}_{2}$	2	2
[36]	2017	Ti6Al4V	Milling	Cryogenic/Dry	0	
[37]	2016	Ti6Al4V	Milling	Cryogenic/Dry	3	3
[38]	2018	Ti6Al4V	Milling	MQL+nanoparticles	1	2
[39]	2016	Ti6Al4V	Milling	Cryogenic/Dry	2	3
[40]	2018	Ti6Al4V	Milling	Cryogenic + MQL	0	2
[41]	2017	Ti6Al4V	Milling	Cryogenic	0	2
[42]	2018	Ti6Al4V	Milling	Ultrasonic vibration-assisted	1	3
[43]	2019	Ti6Al4V	Milling	Cryogenic LN${}_{2}$	0	2
[44]	2018	Ti6Al4V	Milling	Laser-assisted	0	1
[3]	2016	Ti6Al4V	Turning	HPC	18	2
[4]	2018	Ti5553	Turning	MQL/HPC	2	1
[8]	2016	Ti6Al4V	Turning	MQCL	13	2
[10]	2015	Ti6Al4V	Turning	Oil on water MQL/MQCL	19	2
[21]	2016	Ti6Al4V	Turning	Cryogenic/MQL/Cryogenic + MQL	14	
[25]	2018	Ti6Al4V	Turning	HPC	0	1
[26]	2017	Ti6Al4V	Turning	HPC	9	1
[45]	2017	Ti6Al4V	Turning	Cryogenic LN${}_{2}$	13	2
[46]	2016	Ti6Al4V	Turning	HPC	12	2
[47]	2017	Ti Grade 2	Turning	MQL	10	1
[48]	2017	Ti6Al4V	Turning	Gaseous nitrogen	7	1
[49]	2017	Ti6Al4V	Turning	Cryogenic + ethanol	6	
[50]	2017	Ti Grade 2	Turning	MQL (R-H vortex tube)	7	2
[51]	2017	Ti6Al4V	Turning	Dry/MQL	6	1
[52]	2017	Ti6Al4V	Turning	MQCL	7	3
[53]	2018	Ti6Al4V	Turning	MQL	6	2
[54]	2018	Ti6Al4V	Turning	MQL	5	2
[55]	2017	Ti6Al4V	Turning	Dry	3	
[56]	2017	Ti6Al4V	Turning	Cryogenic LN${}_{2}$ (single and dual)	5	3
[57]	2019	Ti6Al4V	Turning	Cryogenic LN${}_{2}$	3	1
[58]	2018	Ti6Al4V	Turning	Specific insert	3	1
[59]	2016	Ti6Al4V	Turning	Cryogenic/MQL/sub zero	2	
[60]	2017	Ti6Al4V	Turning	Cryogenic LN${}_{2}$	3	2
[61]	2018	Ti5553	Turning	Cryogenic/MQL/HPC	3	2
[62]	2018	Ti6Al4V	Turning	Cryogenic LN${}_{2}$	2	2
[63]	2018	Ti6Al4V	Turning	Cryogenic LN${}_{2}$	2	2
[64]	2017	Ti6Al4V	Turning	Cryogenic LN${}_{2}$/N${}_{2}$	2	2
[65]	2017	Ti6Al4V	Turning	MQL	2	2
[66]	2019	Ti6Al4V ELI	Turning	MQCL	1	1
[67]	2018	Ti6Al4V	Turning	Sub-zero HPC	0	1
[68]	2018	Ti6Al7Nb	Turning	MQL	1	2
[69]	2017	Ti6Al4V	Turning	Nanolubrication	1	2
[70]	2018	TC17	Turning	MQL/oil on water MQL	0	1
[71]	2019	Ti5553	Turning	Cryogenic LN${}_{2}$/CO${}_{2}$	0	1
[72]	2019	Pure titanium	Turning	Cryogenic + MQL	0	2
[73]	2018	Ti6Al4V	Turning	MQL/U-CMQL	0	2
[74]	2018	Ti6Al4V	Turning	MQL and MQCL	0	2
[75]	2019	Ti6Al4V ELI	Turning	HPC	0	2
[76]	2018	Ti6Al4V	Turning	Cryogenic LN${}_{2}$/CO${}_{2}$	0	3
[77]	2017	Ti6Al4V	Turning	EHVSL	0	3
[78]	2018	Ti6Al4V	Turning	Cryogenic LN${}_{2}$ (Internal cooling)	0	4
[79]	2018	Ti6Al4V	Turning	Laser-assisted	0	

**Table 3:** Main variables studied in papers on drilling.

Reference	Cutting Force	Cutting Temperature	Surface Integrity	Chip Morphology	Specific Cutting Energy	Tool Wear	Tool Life	Friction Coefficient	Power Consumption
[6]	x			x		x			
[30]	x		x			x			
[31]						x	x		
[32]		x				x			

**Table 4:** Main variables studied in papers on milling.

Reference	Cutting Force	Cutting Temperature	Surface Integrity	Chip Morphology	Specific Cutting Energy	Tool Wear	Tool Life	Friction Coefficient	Power Consumption
[1]	x					x			
[2]			x		x	x	x		x
[9]	x			x		x			
[17]		x	x	x		x		x	
[22]						x	x		
[27]		x				x	x		
[29]	x	x							
[33]	x	x				x			
[34]	x			x		x		x	
[35]		x				x			
[36]	x		x			x			x
[37]					x	x			x
[38]	x	x	x			x			
[39]			x			x	x		
[40]	x		x						
[41]	x		x						
[42]						x			x
[43]			x			x	x		
[44]	x				x				

**Table 5:** Main variables studied in papers on turning.

Reference	Cutting Force	Cutting Temperature	Surface Integrity	Chip Morphology	Specific Cutting Energy	Tool Wear	Tool Life	Friction Coefficient	Power Consumption
[3]			x			x	x		
[4]	x		x	x					
[8]	x		x			x			
[10]	x	x	x	x		x			
[21]			x		x	x	x		
[25]			x	x		x	x		
[26]	x	x	x	x		x			
[45]	x		x						
[46]	x	x		x					
[47]	x		x			x			
[48]			x			x			
[49]	x			x		x			
[50]	x		x			x			x
[51]	x		x			x	x		x
[52]	x		x			x			
[54]			x	x					
[55]	x					x			
[56]		x	x	x	x	x	x		
[57]	x	x	x		x				
[58]		x	x	x		x			
[59]	x	x		x		x	x		
[60]	x		x			x			
[61]	x	x		x		x			
[62]				x					
[63]	x		x	x					
[64]						x			
[65]	x		x			x			
[66]		x	x	x		x			
[67]	x	x		x		x			
[68]	x		x	x	x	x		x	
[69]			x			x			x
[70]	x					x			
[71]	x	x	x	x	x	x			
[72]	x		x			x			x
[73]	x		x	x		x			
[74]					x	x			
[75]	x			x					
[76]			x						
[77]	x		x			x			
[78]		x							
[79]	x								

**Table 6:** Selected papers on milling.

Reference	Journal	Title	Year of Publication
[34]	The International Journal of Advanced Manufacturing Technology	*Effect of cryogenic treatment on the microstructure and the wear behavior of WC-Co end mills for machining of Ti6Al4V titanium alloy*	2017
[17]	Tribology International	*On the benefits of sub-zero air supplemented minimum quantity lubrication systems: An experimental and mechanistic investigation on end milling of Ti6Al4V alloy*	2017
[22]	The 50th CIRP Conference on Manufacturing Systems. Procedia CIRP	*Investigation of the Influence of CO${}_{2}$ Cryogenic Coolant Application on Tool Wear*	2017
[9]	International Journal of Precision Engineering and Manufacturing	*Milling of titanium alloy with cryogenic cooling and minimum quantity lubrication (MQL)*	2017
[2]	Machining Science and Technology	*Comparative investigation on using cryogenic machining in CNC milling of Ti6Al4V titanium alloy*	2016
[1]	Journal of Mechanical Science and Technology	*The effect of cryogenic cooling and minimum quantity lubrication on end milling of titanium alloy Ti6Al4V*	2015
[29]	International journal of advanced manufacturing technology	*Determination of optimal laser power according to the tool path inclination angle of a titanium alloy workpiece in laser-assisted machining*	2016
[27]	Wear	*Tool life and wear mechanisms in laser assisted milling Ti6Al4V*	2015

**Table 7:** Selected papers on turning.

Reference	Journal	Title	Year of Publication
[3]	The International Journal of Advanced Manufacturing Technology	*High-pressure coolant on flank and rake surfaces of tool in turning of Ti6Al4V: investigations on surface roughness and tool wear*	2016
[47]	Journal of Cleaner Production	*Sustainable machining of aerospace material- Ti (grade 2) alloy: modelling and optimization*	2017
[48]	Journal of Manufacturing Processes	*Temperature effects on the Ti6Al4V machinability using cooled gaseous nitrogen in semi-finishing turning*	2017
[45]	The International Journal of Advanced Manufacturing Technology	*Study of surface roughness and cutting forces using ANN, RSN, and ANOVA in turning of Ti6Al4V under cryogenic jets applied at flank and rake faces of coated WC tool*	2017
[49]	CIRP Journal of Manufacturing Science and Technology	*Increasing efficiency of Ti-alloy machining by cryogenic cooling and using ethanol in MRF*	2017
[8]	The International Journal of Advanced Manufacturing Technology	*An experimental investigation on effect of minimum quantity cooling lubrication (MQCL) in machining titanium alloy (Ti6Al4V)*	2016
[10]	The International Journal of Advanced Manufacturing Technology	*Tool wear in Ti6Al4V alloy turning under oils on water cooling comparing with cryogenic air mixed with minimal quantity lubrication*	2015
[21]	48th CIRP Conference on Manufacturing Systems – CIRP CMS 2015. Procedia CIRP	*Investigation of cooling and lubrication strategies for machining high-temperature alloys*	2016

**Table 8:** Selected papers on drilling.

Reference	Journal	Title	Year of Publication
[32]	Materials and Manufacturing Processes	*Temperature rise in workpiece and cutting tool during drilling of titanium aluminide under sustainable environment*	2018
[6]	Journal of Cleaner Production	*Environmentally friendly drilling of intermetallic titanium aluminide at different aspect ratio*	2017
[30]	Precision Engineering	*Micro-drilling of Ti6Al4V alloy: The effects of cooling/lubricating*	2016
[31]	16th Machining Innovations Conference for Aerospace Industry	*Effect of water oil mist spray (WOMS) cooling on drilling of Ti6Al4V alloy using Ester oil based cutting fluid*	2016
